# Descriptive vs. inferential cheating

**DOI:** 10.3389/fpsyg.2013.00627

**Published:** 2013-09-11

**Authors:** David Trafimow

**Affiliations:** Department of Psychology, New Mexico State UniversityLas Cruces, NM, USA

**Keywords:** descriptive cheating, inferential cheating, null hypothesis significance testing procedure, cheating in psychology, false null hypotheses

Given the recent and highly publicized scandals involving psychology researchers who cheated, the proliferation of articles on related topics is unsurprising. As an example, Simons et al. (2011) pointed out subtle ways in which researchers can increase their false positive rate above the nominal level of *p* < 0.05. From my perspective, a major limitation of the literature on cheating has been a failure to distinguish between two kinds of cheating (bias might be a kinder word), that I term *descriptive* and *inferential* cheating. I intend to demonstrate that inferential cheating is not as destructive as descriptive cheating.

So what is descriptive and inferential cheating? Descriptive cheating involves the false reporting of descriptive data, such as sample means, proportions, standard deviations, and so on. The harm of descriptive cheating is obvious, has been demonstrated by previous scandals, and needs no further elaboration here. In contrast, when a researcher cheats inferentially, the descriptive data are true but the reported *p*-values (and associated *t*-tests, *F*-tests, and so on) are not. My conclusion that inferential cheating causes only limited harm is based on demonstrations that the null hypothesis significance testing procedure (NHSTP) is invalid. My conclusion is that although providing false information that matters a lot, such as wrong descriptive statistics, can do much harm, providing false information that matters hardly at all, such as false *p* values, does not do much harm.

So what is wrong with the NHSTP? The basic idea is that if we are to reject the null hypothesis, it should be shown to have a low probability of being true, given the finding. But a *p*-value does not provide this; rather, a *p*-value only shows that a finding is rare given the null hypothesis (Nickerson, 2000). As Kass and Raftery (1995) pointed out, knowing that a finding is rare given a hypothesis is not useful unless one knows how rare the finding is given a competing hypothesis. Also, Trafimow (2003) demonstrated that (1) the null hypothesis can have a very high probability (including a probability of 1) of being true even when *p* < 0.05, (2) *p*-values generally are inaccurate estimators of probabilities of null hypotheses, and (3) the conditions needed to make *p*-values valid indicators of probabilities of null hypotheses preclude the researcher from gaining much information from the NHSTP. Furthermore, Trafimow and Rice (2009) demonstrated that the correlation between *p* values and probabilities of null hypotheses is low to begin with, and decreases to triviality when dichotomous “accept” or “reject” decisions are made based on cutoff numbers such as 0.05 or 0.01.

The famous theorem by Bayes provides examples whereby the null hypothesis will be rejected even when it has a strong likelihood of being true. Suppose that the prior probability of the null hypothesis is 0.95, the probability of the finding given the null hypothesis is the traditional value of 0.05 (so the null hypothesis is rejected), and the prior probability of the finding given that the null hypothesis is not true is 0.06. In that case, the posterior probability of the rejected null hypothesis is $\frac{\left(0.95)(0.05\right)}{\left(0.95)(0.05)+(0.06)(1-0.95\right)}=0.94$.

In the foregoing example, I tacitly allowed the null hypothesis to represent a range of values. Worse yet, however, in most empirical psychology articles, the null hypothesis refers to a single value (e.g., that the difference between two conditions is zero). But when the null hypothesis refers to a specific value, it is a practical certainty that the value is not exactly true. With an infinite number of possible values, the probability that the single value specified by the null hypothesis is exactly true approaches zero (e.g., Meehl, 1967; Loftus, 1996; Trafimow, 2006), and so it should be rejected.

The NHSTP has been demonstrated to be invalid and it results in *p*-values that have little correlation with actual probabilities of null hypotheses. We also have seen that when the null hypothesis specifies a point, as opposed to a range, it is almost certainly false regardless of the obtained *p*-value. Thus, whether the null hypothesis specifies a range or a point, the NHSTP is invalid. Arguably, because of its invalidity, the NHSPT should not be performed, and so inferential cheating bypasses a procedure that should not be used anyway. Thus, where is the harm in avoiding the use of a procedure that is blatantly invalid and only trivially correlated with what we really need to know (the probabilities of null hypotheses)?

Let me be clear about what I am not saying. First, I am not disagreeing with various prescriptions for avoiding inferential cheating, particularly because many of them would reduce descriptive cheating too, and the latter is much more important. Second, I am not arguing that all inferential cheating is harmless; for example, harm can result when one makes improper estimates of population parameters based on poor inferential procedures even with accurate sample statistics. Third, it is quite possible that in attempting heroic measures to obtain *p* < 0.05, descriptive statistics also might be influenced, and this would be harmful to psychology. Fourth, from a deontological point of view, cheating is unethical in its own right, even apart from specific demonstrable consequences, and so the present argument should not be taken as a justification for any cheating whatsoever.

With the foregoing caveats in place, my main point is as follows. Although descriptive cheating is harmful in specific and demonstrable ways, this is not true of the most common type of inferential cheating, which results in the rejection of null hypotheses in ways that deviate from ostensible proper practice. Clearly such inferential cheating is undesirable in a general deontological sense, but it is difficult to enumerate specific consequential harm to the field of psychology. That specific consequential harm from inferential cheating is so difficult to enumerate perhaps constitutes a further argument that the NHSTP should not be required for publication.
